# HelmCoP: An Online Resource for Helminth Functional Genomics and Drug and Vaccine Targets Prioritization

**DOI:** 10.1371/journal.pone.0021832

**Published:** 2011-07-08

**Authors:** Sahar Abubucker, John Martin, Christina M. Taylor, Makedonka Mitreva

**Affiliations:** 1 The Genome Institute, Washington University School of Medicine, St. Louis, Missouri, United States of America; 2 Department of Genetics, Washington University School of Medicine, St. Louis, Missouri, United States of America; The George Washington University Medical Center, United States of America

## Abstract

A vast majority of the burden from neglected tropical diseases result from helminth infections (nematodes and platyhelminthes). Parasitic helminthes infect over 2 billion, exerting a high collective burden that rivals high-mortality conditions such as AIDS or malaria, and cause devastation to crops and livestock. The challenges to improve control of parasitic helminth infections are multi-fold and no single category of approaches will meet them all. New information such as helminth genomics, functional genomics and proteomics coupled with innovative bioinformatic approaches provide fundamental molecular information about these parasites, accelerating both basic research as well as development of effective diagnostics, vaccines and new drugs. To facilitate such studies we have developed an online resource, HelmCoP (Helminth Control and Prevention), built by integrating functional, structural and comparative genomic data from plant, animal and human helminthes, to enable researchers to develop strategies for drug, vaccine and pesticide prioritization, while also providing a useful comparative genomics platform. HelmCoP encompasses genomic data from several hosts, including model organisms, along with a comprehensive suite of structural and functional annotations, to assist in comparative analyses and to study host-parasite interactions. The HelmCoP interface, with a sophisticated query engine as a backbone, allows users to search for multi-factorial combinations of properties and serves readily accessible information that will assist in the identification of various genes of interest. HelmCoP is publicly available at: http://www.nematode.net/helmcop.html.

## Introduction

Recent advances in next-generation massively parallel sequencing platforms [1] have made it possible to sequence the genomes of various pathogens at a fraction of earlier costs. This rapid increase in the availability of millions of DNA sequence reads in a short time will have a huge impact on research of eukaryotic pathogens including Neglected Tropical Diseases (NTD) [2]. Comparative genomics is the dominant approach to organize, interpret, and utilize the vast amounts of the sequence information anticipated from these pathogens, however it is a challenging task to tackle the amount of genome related information available and therefore user friendly resources that can organize this data in a usable manner are necessary.

Helminth parasites are parasitic worms from the phyla Nematoda (roundworms) and Platyhelminthes (flatworms). Helminthes are a very diverse species that have adapted to many different ecosystems and can be free-living, endosymbionts or parasites. Parasitic worms from the phyla Nematoda cause a range of diseases in humans including ascariasis, trichuriasis, ancylostomiasis, filariasis, toxocariasis and trichinosis. The effects range from serious and often disfiguring conditions, such as elephantiasis and blindness, to the more subtle effects on child development, pregnancy and productivity, resulting in maintenance of poverty in many developing countries. Schistosomiasis is the most prevalent platyhelminth caused NTD, and possibly associated with increased horizontal transmission of HIV/AIDS [3]. Approximately 85% of the NTD burden in sub-Saharan Africa results from helminth infections [4]. The collective burden of the common helminth diseases (over 2 billion human infections; [5]) rivals that of main high-mortality conditions such as HIV/AIDS or malaria. In addition, vaccine efficacy for other disease conditions could also be adversely affected by nematode infections [6]. Plant and animal parasitic nematodes damage crops, affect agricultural productivity, cause diseases in livestock and result in losses of billions of dollars. Many classes of anthelmintics are used to treat helminth infections, however, rise in resistance to these treatments [7], [8] confer an urgent need to gain insight into nematode parasitism adaptation and to develop new control strategies.

In the next five years, projects at the Washington University's Genome Center (http://www.genome.gov/10002154) and the Wellcome Trust Sanger Institute (http://www.sanger.ac.uk/Projects/Helminths/) will increase the available sequence data on human helminthes and their close relatives by an order of magnitude, adding more than 20 draft genomes to the list of existing genomes, making the helminthes one of the most extensively sequenced eukaryotic pathogens and creating an invaluable resource for comparative genomic studies. While intensive research that will result in identification of many targets for development of new drugs, vaccines and diagnostics is expected to be conducted, there is no reported central resource that allows storage, annotation, comparison and analysis of helminth proteomes with a focus on exploring supportive information that will help prioritize such targets. To address this, we have developed the HelmCoP resource (Helminth COntrol and Prevention), an online portal that integrates functional genomic and molecular data from free-living and parasitic worms, in addition to human, animal and plant hosts, to expedite identification of drug, pesticide, and vaccine targets. HelmCoP is the first comprehensive database for performing comparative genomic analysis and target prioritization, with a focus on nematodes and platyhelminthes. Host genomes are included to identify well-conserved orthologies to the pathogens. It can also be used as an initial screen to deduce off-target effects, though further analysis will have to be performed to fully quantify these effects. Several model organisms are also included to leverage available resources for comparative analyses. This also allows bioinformatics analysis and comparison of various targets across multiple species and/or phyla.

HelmCoP has an extensive catalog of various supporting information to increase confidence in results and to aid in conceiving and planning subsequent experiments. The HelmCoP interface allows identification of genes of interest, enables comparisons based on function, structure, sequence similarity, orthology and stage-specific expression and retrieves collections of data with required characteristics. Helminth proteomes are associated with several data types including expression, developmental information, pre-computed similarity searches to multiple annotation databases, secondary-structure information, assignment of ontologies to gene products, and metabolic pathway associations. Utilizing information from DrugBank [9], HelmCoP identifies available compounds, which bind to targets similar to those that bind to known drugs, while also searching for targets with a user-defined criteria. The portal has the versatility to enable the user to search for drug targets for specific helminth or group of helminth pathogens and also allow the user to search for broad-spectrum drug targets that span multiple species. In addition, features associated with successful vaccine products are also included as a separate search function to assist in identifying potential vaccine candidates. HelmCoP integrates all this information for effective data mining for comparative genomics, with the aim of providing a bridge between molecular and genomic data with clinically relevant information that can be used to expedite drug and drug target discovery, as well as vaccine development.

## Results

**Figure 1:**
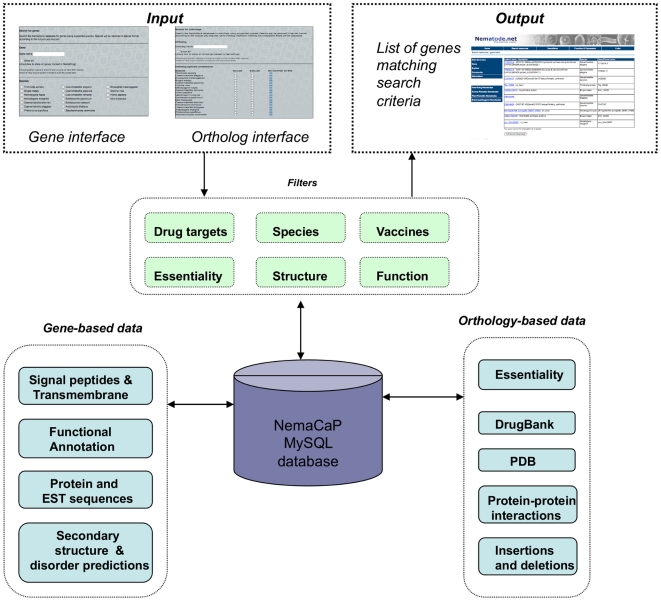
Schematic representation of HelmCoP. Two input interfaces are available enabling the user to search based on individual genes or orthologous groups, i.e. to find genes that are present in one species or more species. The results can be filtered based on the user-defined criteria. There is also gene-based and orthology-based data that can be output. The filters and output data options are sent through the MySQL database, and all the information regarding the genes is displayed in a table format on an output screen.

The HelmCoP search portal, an independent component of Nematode.net [10], has been developed with the aim of integrating data from different sources for all available nematode and platyhelminth genomes and sharing it through an effective and useful interface. It has been designed with two primary goals - i) to enable comparative functional genomics and ii) to prioritize drug and vaccine targets for intervention strategies. HelmCoP features a rich set of functionalities with robust integration of data from both internal and external sources, allowing the user to make relevant connections between genomic information from different nematodes and playthelminthes, hosts and other model organisms. A comprehensive schema, designed to support efficient data mining from the large volume of disparate information, is comprised of multiple entities, each representing a feature related to a gene (Figure 1). All sequence-related information available for each organism in HelmCoP is listed in Table 1. Annotations to domain, ontology, and enzyme databases along with expression data and associations to DrugBank targets and PDB structures [11], are available for the nematodes and platyhelminthes. Secondary and disordered structure predictions are available only for the nematodes.

**Table 1:** List of species in HelmCoP and their associated annotations.

*Species*	*#Proteins*	*Orthology* a	*DrugBank* b	*Structure* c	*Functional annotation* d	*Expression* e	*Secondary Structure* f	*Indels* g
***Nematodes***								
*Brugia malayi*	11,610	✓	✓	✓	✓	✓	✓	✓
*Caenorhabditis elegans*	24,054	✓	✓	✓	✓	✓	✓	✓
*Caenorhabditis brenneri*	30,702	✓	✓	✓	✓	✓	✓	
*Caenorhabditis briggsae*	21,978	✓	✓	✓	✓	✓	✓	
*Caenorhabditis japonica*	25,870	✓	✓	✓	✓	✓	✓	
*Caenorhabditis remanei*	31,518	✓	✓	✓	✓	✓	✓	
*Meloidogyne hapla*	14,421	✓	✓	✓	✓	✓	✓	✓
*Meloidogyne incognita*	20,359	✓	✓	✓	✓	✓	✓	✓
*Pristionchus pacificus*	29,201	✓	✓	✓	✓	✓	✓	
*Trichinella spiralis*	16,124	✓	✓	✓	✓	✓	✓	✓
***Platyhelminthes***								
*Schistosoma mansoni*	11,789	✓	✓	✓	✓	✓		✓
*Schistosoma japonicum*	13,469	✓	✓	✓	✓	✓		✓
***Host/Outgroups***								
*Arabidopsis thaliana*	28,952	✓						
*Drosophila melanogaster*	21,921	✓						
*Homo sapiens*	76,592	✓						
*Glycine max*	46,430	✓						
*Mus musculus*	50,068	✓						
*Saccharomyces cerevisiae*	6,717	✓						

a Ortholog groups were generated from all 18 species through OrthoMCL;

b Drugbank annotation includes targets with homology to proteins in HelmCoP, compound information and cheminformatic data;

c Structural information includes signal peptide and transmembrane spanning information, homology to PDB, and presence of a coiled coil;

d Functional annotation includes Gene Ontology (GO), KEGG Orthology (KO), and InterPro domains;

e Expression is based on stage/tissue of origin of Expressed Sequence Tags (EST);

f Secondary structure includes secondary and disorder predictions;

g Indels relative to non-nematode proteins are included for a subset of genomes;

HelmCoP has two search interfaces, one centered on individual genes and the other serving orthology-based searches queries. The search criteria available from both interfaces are similar, with some features unique to each. The gene page is focused on either a specific gene, or genes satisfying the required filters and the orthology search page is focused on lists of genes that satisfy the filters in addition to the required orthology criteria (Figure 1). There is also a WU-BLAST 2.0 [12] search page that allows users to search their own sequences against the HelmCoP proteomes using blastp (for protein queries) or blastx (for nucleotide queries). The results of the WU-BLAST search are sent to the user *via* email, which is collected by HelmCoP at the beginning of the search. This is done to allow users to enter a large number of sequences to HelmCoP and not have to wait while the WU-BLAST search is performed. The protein targets identified by this search can then be used as input in either of the two search interfaces. The search parameters and output options are discussed in the following sections.

### Searchable Parameters

#### Gene / Orthologous groups

**Figure 2:**
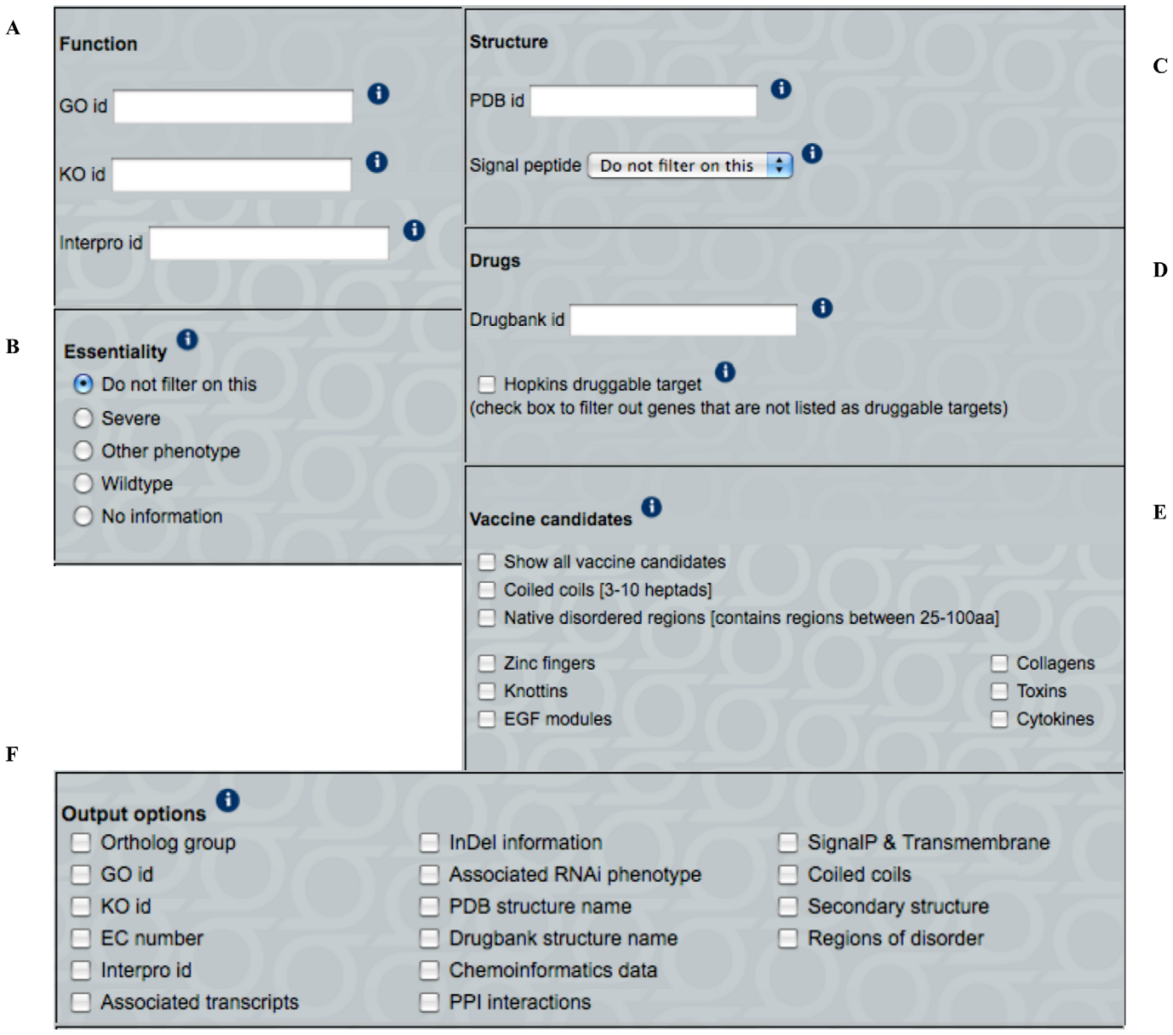
HelmCoP contains two main search interfaces. Both main search functions contain the following filtering options: (A) Function search, (B) RNAi phenotype search, (C) PDB and Signal Peptide search, (D) DrugBank target search, and (E) Vaccine targets search. (F) Output options that can be added to any search to provide additional information regarding the proteins to aid in user-defined prioritization and selection for subsequent testing. Additional information regarding usage is available by clicking on the info boxes (represented by (i)) throughout the page.

One of the salient features of the HelmCoP resource is the ability to rapidly access orthology and homology for genes of interest from the most comprehensive set of helminth proteomes available. Species required to be present in an orthologous group and species that are to be excluded can be selected using the orthology portal page. With such a large number of worm genomes, orthologous groupings can be useful for isolating groups of genes that are only found in certain organisms but not in others, while also providing annotation information. Annotation may only be available for a few proteins within the orthologous group and can be extrapolated for proteins within the group that lack annotations. This feature is extremely useful for comparative genomics studies. Determining orthologous genes in other species can also shed insights into the evolution of the gene and offer additional clues about its functional profile, including gene duplications, deletions or insertions. This ability is extremely powerful for both comparative genomics and drug target discovery. Alternatively, the genes can be searched by individual genes names or species using the gene page portal and orthologous group information can be an output option (Figure 2). Orthology was inferred using OrthoMCL [13], which generated a total of 38,776 orthologous groups from the 18 species, and 486 groups included all 18 species. OrthoMCL was chosen to identify orthology based on evaluations using functional annotations that we have performed earlier (data not shown) and also based on comparisons to other orthology programs [14]. Table 2 displays counts of genes and groups built from specific species groupings and Table S1 lists details of groups generated from all combinations of the HelmCoP organisms.

**Table 2:** Selected ortholog groups represented in HelmCoP.

*Group (# of Species)*	*Proteins*	*Ortholog Groups* a
All NemaCaP species (18)	43,204	486
Nematodes (10)	967	44
Plant parasitic nematodes (2)	3,190	1,004
Animal parasitic nematodes (2)	141	37
Parasitic nematodes (4)	32	5
Nematodes and Platyhelminthes (12)	80	4

a The groupings represent groups and genes from only species that has at least one gene in the group.

#### Functional Annotation

The various genomes can be parsed based on several functional features, including Gene Ontology (GO; [15]), KEGG Orthology (KO; [16]), and InterPro domains [17] (Figure 2A). GO annotations provide a controlled vocabulary for comparing proteins from the different pathogens and their hosts with the three organizing principles (cellular component, biological process, and molecular function). For instance, by selecting a GO term and one or more helminth species, all the genes from these species annotated with that GO term would be displayed in the output. In addition, multiple output options could also be chosen to determine if the protein had homology to the PDB or if there was a DrugBank target that was similar, etc. Interpro identifiers provide protein “signatures” that can be used to compare proteins in the different helminth proteomes. The KO numbers represent groupings of genes with similar functions and can be mapped to pathways (KEGG Pathways) and drugs (KEGG Drug) within the KEGG database. These annotations are also output options and can be also be used without any filtering parameter.

#### Essentiality

RNA interference (RNAi) has greatly accelerated the rate of functional characterization of genes. RNAi was applied in *C. elegans* to screen individual genes, and then adapted for high-throughput screening of gene sets from whole chromosomes [18], [19], cDNA collections [20] and ultimately genome-wide clone sets [21], [22], [23]. As of today, RNAi information is available for nearly 20,000 *C. elegans* genes (96% of molecular loci). While the mechanism responsible for the RNAi response is evolutionarily conserved in helminthes, the ‘spreading’ of RNAi to give a systemic response to the localized introduction of dsRNA is limited to a few species. In addition, applying RNAi to parasitic helminthes poses significant challenges due to the complexity of obligate parasitic life cycles, with movement into and out of the host, makes both the delivery of dsRNA and the assessment of phenotype difficult. RNAi has been documented for a limited number of genes in parasitic helminthes (reviewed in [24], [25], [26]). In several parasitic helminthes, there is a strong correlation between sequence conservation of a parasite gene and its *C. elegans* homolog and the presence of a RNAi phenotype in *C. elegans* [27], [28]. Therefore it is possible to extrapolate from *C. elegans* RNAi phenotypes to understand which orthologous genes might have crucial roles in other helminthes, including parasites where high-throughput screening is not yet possible. However, it should be noted that lethality in *C. elegans* might not necessarily indicate lethality in other organisms as the RNAi pathway might be different and hence, this caveat must be considered when using the RNAi lethality filter.

In HelmCoP, filtering based on RNAi data enables the user to find genes in species other than *C. elegans* that may be essential and thus, good proteins to target for drug discovery. HelmCoP can be parsed for proteins in other species that have orthologous *C. elegans* genes that exhibit RNAi phenotypes (Figure 2B). HelmCoP classifies RNAi phenotypes based on severe, wild type, other phenotype, and no information. The associated RNAi phenotypes can also be output with any general search.

#### Structural Annotation

Signal peptide predictions are searchable for all the organisms (Figure 2C). Secreted or excreted protein products are involved in protein targeting and, though these signals trigger cellular events, some of these proteins could potentially be involved in host-parasite interactions, which could yield important insights regarding how the parasite evades the immune system of the host. Therefore, proteins with signal peptides may be attractive drug or vaccine targets. Signal peptide information can be augmented by choosing the associated transcripts option in the output. If there is associated transcript information, the stage or tissue where the transcript is expressed could provide clues about the importance of this protein for parasitism. Signal peptide information can also be output for all genes by selecting the output option.

Transmembrane regions are also searchable, as transmembrane receptors are often involved in signal transduction and major classes of membrane receptors, like G-protein coupled receptors, ion-channel receptors, etc., have been targets of almost 40% of the available drugs on the market, making these proteins important for discovering new therapies [29].

A solved crystal structure is a valuable asset when classifying proteins, determining structure and function, and designing drugs. Unfortunately, many nematode proteins lack a solved X-ray crystal or nuclear magnetic resonance (NMR) structure. For proteins that do not have a solved crystal structure, homology models can be generated from proteins in the PDB and therefore, PDB structures similar to HelmCoP proteins are also stored. Entering a specific PDB identifier (Figure 2C) and choosing species of interest will return proteins in the various species that have homology to that PDB structure. Alternatively, homology to the PDB can be output in any general search.

#### Drugs and Drug Targets

HelmCoP can be searched based on DrugBank IDs (Figure 2D), and DrugBank entries with homology to proteins in the selected species can be retrieved. DrugBank contains chemical, pharmacological, and pharmaceutical information of drugs, as well as drug targets and their associated sequence. The drugs range from FDA-approved to experimental drugs, as well as nutraceuticals. For instance, if a particular drug is known to be effective for a different parasite, the DrugBank ID could be searched in HelmCoP and helminth species that have similar targets will be displayed.

A link to the DrugBank drug cards entry is provided, as well as cheminformatic information that can be used to evaluate the quality of the drug. The drug cards include information regarding the status of the compound (approved, experimental, etc), current indication for the drug, and drug synonyms are also included in this output. The output includes parameters determined by Lipinski et al. 2001 [30] that can be used to evaluate drug potential and to also determine if the drug will be orally active in humans (absorption, distribution, metabolism, and excretion - ADME). For example, in this report the following parameters were found in compounds that became successful pharmaceuticals: LogP≤5, number of hydrogen bond donors ≤5, number of hydrogen bond acceptors ≤10, and the molecular weight ≤500 Da. As HelmCoP also includes plant parasites, the cheminformatic information can also be used for pesticide discovery. Different parameters have been found to be specific for finding good agrochemicals [31]: molecular weight ≤500; LogP≤5; hydrogen-bond donors ≤3; and hydrogen-bond acceptors ≤12. The cheminformatic information can be downloaded, and compounds can then be ranked and prioritized based on user-defined cheminformatic criteria.

The Simplified Molecular Input Line Entry System (SMILES) [32] string, which can represent a structure concisely via an ASCII string, is also listed in the cheminformatic output and can be used to find molecules with similar properties. Finding similar molecules allows a Structure Activity Relationship (SAR) study to be conducted, as various programs will search a database using the SMILES string along with a Tanimoto Index Similarity score cutoff (PubChem, ChemViz).

Proteins that bind to drugs that follow Lipinski's rule of 5 are considered ‘druggable’ by Hopkins et al, 2002 [33]. The proteins fall into six main categories defined by Interpro domains: G-protein coupled receptors (GPCRs), serine/threonine and tyrosine protein kinases, zinc metallopeptidases, serine proteases, nuclear hormone receptors, and phosphodiesterases. HelmCoP has an option to filter all proteins in selected species that are “druggable” by Hopkins' criteria (Figure 2D).

#### Vaccine candidates

Epitopes are regions of a protein that are recognized by the immune system, which can be linear and unstructured or have a defined structural motif. The fundamental premise of the epitope-based approach to vaccine development involves improvement of the responses induced by the natural immunogen when the response is not optimal. This is achieved by isolation or optimization of specific components of the response. Structural domains in selected proteomes can be mapped onto a small mini-protein and used as a vaccine [34]. Alpha-helical coiled coils have been identified as potential vaccine candidates using bioinformatic techniques where antibodies specific to the coiled coil peptide reacted with parasitic proteins and also inhibited in vitro parasite growth [35]. Unstructured regions have been recognized in sera and blood mononuclear cells of residents in areas where malaria is prevalent [36]. Parts of EGF domains in *Plasmodium vivax* are being considered for transmission blocking vaccines [37]. These domains can be filtered using HelmCoP (Figure 2E). Regions of disorder can also be filtered through HelmCoP and potentially used as vaccine candidates. Using this filter, proteins in various genomes that meet the criteria for structural domains and regions of disorder could be prioritized and tested as potential vaccine targets. Other features like signal peptides or RNAi could be used in conjunction with the vaccine search to narrow down the list of candidates for prioritization.

### Output Options for User-Defined Prioritization

Output results are displayed in a table format and additional information can be obtained by clicking on the provided links. Most of the information returned by HelmCoP contains links directly to the database's own website. This way, the latest entries for the annotations will be available to the user. Results are also available for download in a delimited text format. The following are output options in HelmCoP that provide additional information for user-defined prioritization (Figure 2F).

#### Enzyme Commission Number

Most enzymes are associated with equivalent Enzyme Commission (EC) numbers, which provide general information about a particular enzyme based on a classification scheme. The KEGG compound database is comprised of chemical substances from biological systems, and a subset of entries has associated enzymes. By linking these enzymes to genes, lists of compounds can be retrieved for genes of interest.

#### Secondary-structure, coil-coils and disorder predictions

In the absence of high-resolution data from X-ray crystallography or NMR, secondary-structure prediction methods are necessary to begin to elucidate tertiary and quaternary structure. Secondary-structure prediction can be combined with other characteristics to recognize a fold that already exists in a protein with which it does not share sequence similarity. Many helminth proteins do not have solved X-ray crystal structures and are not homologous to proteins in the PDB, therefore the systematic secondary-structure predictions we provide would aid in determining protein folds and provide a starting place for understanding and modeling tertiary and quaternary structure. Secondary-structure prediction programs rely heavily upon datasets from which they were built, so it is necessary to use several different programs to have improved characterization of a protein's secondary and disorder structure. We compiled results for three secondary-structure prediction methods for the entire collection of helminth genes.

Coiled coils are common structural motifs found in all organisms, consisting of 2 or more intertwined alpha-helices. The heptad repeat is the hallmark of coiled coils, which consists of a repeating pattern **a**,**b**,**c**,**d**,**e**,**f**,**g**, where **a** and **d** are hydrophobic residues and **e** and **g** are charged residues. This motif is important for mediating protein-protein interactions, oligomerization, membrane fusion, and biological processes such as regulation of gene expression through various transcription factors. Coiled-coil regions were identified in all the nematode genomes and can be displayed by selecting the output option.

Disordered or unstructured protein regions for genes could be relevant in determining function and structures, as these genes have been shown to be important for molecular recognition and cell regulation [38]. Many proteins or regions of proteins that are unfolded in their native state have been found to be involved in molecular recognition, often undergoing disordered-to-ordered transitions to fold and form complexes with other proteins [39]. These interactions are typically highly specific, but have weak binding affinity. Accurately identifying disordered regions can be useful when designing drugs that target a protein [40] and also when determining the structure via X-ray crystallography. HelmCoP provides a graphical illustration of the disordered regions for each nematode protein separately.

#### Pathogen specific insertions and deletions

Another avenue in utilizing orthology information is to seek amino acid insertions and deletions (indels) that characterize a specific group of pathogens but are absent from host organisms. These indels can serve as unique indel-based targeting for controlling the specific group of helminthes. Indels that are located in proteins essential to the survival of the specific group of pathogens can be candidates for high-efficacy drug targets (e.g. [41], [42]; Table S2).

#### Protein-Protein Interactions

Proteins rarely act in isolation and often interact with other proteins to fulfill their biological function, which results in complex protein-protein interaction (PPI) networks. Discovery of important PPIs could lead to the next frontier of drug targets for parasitic nematodes. Despite PPIs being challenging drug targets, there have been recent successes with targeting PPI interactions by small molecules (e.g. [43]). Interactions between host and parasite proteins point to biological functions and are part of PPI networks. The MINT [44] and IntAct [45] PPI databases are associated with genes from *C. elegans*, *H. sapiens*, *D. melanogaster*, *S. cerevisae*, *A. thaliana*, and *M. musculus* and are then linked to the genes in HelmCoP through orthology.

#### Associated Transcripts (EST/cDNAs)

Expression data based on Expressed Sequence Tags (EST) are available for the helminthes in HelmCoP (Table S3). Expression data gives additional support for the genes of interest by providing information on which stages, genders, or tissue types the gene is expressed in and also provides abundance profiles. Knowledge of stage or tissue specific expression is vital in drug design or vaccine target studies, and in comparative genomics. HelmCoP displays links to EST entries in GenBank for the genes selected, if they have EST support.

### Applications of HelmCoP

The primary goals in developing HelmCoP are to provide a suitable platform for comparative functional genomics and to prioritize drug and vaccine targets for control and prevention of helminth infections. Here we provide several examples, which illustrate the powerful search potential of HelmCoP in these two areas.

#### Comparative Genomics

**Figure 3:**
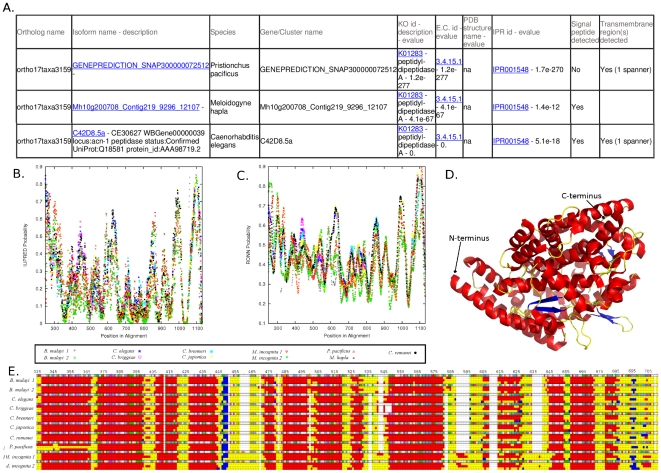
Comparative genomics of ortholog group, ortho17taxa3159. (A). Truncated output of the HelmCoP search (the complete search can be found in Table S4). Alignment of IUPRED (B) and RONN (C) disorder prediction programs. The disorder of the following proteins from ortho17taxa3159 were plotted: 14954.m01684 (*B. malayi 1*), 14954.m01756 (*B. malayi 2*), C42D85.a (*C. elegans*), CBG14607 (*C. briggsae*), CBN25768 (*C. brenneri*), CJA07982 (*C. japonica*), prot_Minc05651 (*M. incognita 1*), prot_Minc10796 (*M. incognita 2*), GENEPREDICTION_SNAP300000072512 (*P. pacificus*), Mh10g200708_Contig219_9296_12107 (*M. hapla*), CRE00266 (*C. remanei*). D. PDB entry 1UZE rendered using Pymol (Version 1.2r3pre) with helices, loops, and β-sheets colored red, yellow, and blue, respectively E. Alignment of the secondary-structure predictions downloaded from HelmCoP using JalView (Waterhouse et al. 2009). The sequences are colored based on clustalw (Thompson et al. 1994). For the secondary-structure prediction, helices are colored red, β-sheets blue, and random coils and loops are colored yellow. The disorder and secondary structure were aligned using in-house PERL script after aligning fasta sequences provided by HelmCoP using MUSCLE (Edgar 2004).

Specific lists of genes restricted to certain taxa, and/or excluding certain taxa can be generated based on orthology. For instance, searching genes shared in animal parasites but absent from all other species returns a list of genes that are specific to the animal parasites. We use the nematode molting cycle as an example to illustrate the functionality of HelmCoP as a comparative genomics tool. There are a number of *C. elegans* genes that are known to be essential for nematode molting and among these are several types of peptidases, including metallo-, serine-, cysteine-, threonine- and aspartic peptidases. Using the most basic GO identifier for peptidases (GO:0006508) and the ortholog search interface, we searched for peptidases that may play a role in molting across all nematodes. In addition to the GO term input, all nematodes species were also selected to be included in the orthology search. This returned 4,180 genes grouped in 110 orthologous groups. Requiring presence of a signal peptide for secretion further filters the list of candidates and returns 42 orthologous groups. Finally, if a severe RNAi phenotype is also required, 19 orthologous groups are returned (Table S4). Entering the ortholog group name on the orthology search page returns all the genes that are in the group along with selected output options. For example, the ortholog group, ortho17taxa3159, contains the *C. elegans* gene C42D8.5, which has been found to be controlled by the nuclear hormone receptors, nhr-23 and nhr-25, and plays an essential role in molting. This group also includes proteins from *H. sapiens*, *D. melanogaster*, *A. thaliana* and *M. musculus*, in addition to all the nematodes. In *H. sapiens* and *M. musculus*, the protein plays a role in catalyzing the conversion of angiotensin I into angiotensin II. In *C. elegans*, this protein lacks the active site for the metallopeptidase [46], but the active site may be present in other nematodes in this ortholog group. HelmCoP outputs structural features, which allows for further comparative structural genomics study (Figure 3A). The two disorder predictions show similar trends for nearly all the proteins in this ortholog group (Figure 3B and C). Only a few proteins in this group showed homology to proteins in the PDB (Figure 3D), however the secondary structure of the proteins are nearly identical (Figure 3E). Despite the lack of homology to the PDB, structural evidence suggests homology modeling may be possible and that the proteins in this group play a role similar to C42D8.5 in the molting process.

Another orthologous group, ortho17taxa3783, contains the *C. elegans* gene Y54E10BR.5, which is a signal peptidase and has been found to be essential for nematode molting [47]. Using the HelmCoP database, further structural comparative genomics was done with the nematode orthologs (Figure S1). The disorder prediction and secondary-structure alignment demonstrate the structural similarity between Y54E10BR.5 and the other proteins in this group, providing structural evidence that these proteins may be important in molting as well. Another enzyme, F08C6.1, which is in a different orthologous group, ortho17taxa1084, has also been found to be essential for molting in *C. elegans* [48]. Further, our search revealed three other *C. elegans* genes that localize to the cuticle but have not been implicated in molting, T03E6.7, C37H5.9, ZC373.1, and several other genes that have not been studied. The search also allows the user to examine orthologous proteins from other species that may have a similar function. Nematode genes from these three ortholog groups are listed in supplemental material online (Table S4). HelmCoP also allows the user to get stage-specific expression information on these proteins. Different combinations of species could be included or excluded in this type of search.

#### Drug Target Discovery

A primary way to identify drugs for repositioning is by using the DrugBank identifiers of known anthelmintics or other antiparasitic drugs; a search using DrugBank identifiers returns a list of all genes that are associated with the specified DrugBank target. For example, quinacrine (DB01103) is used as an anthelmintic drug to treat platyhelminth tapeworm infections. It is also an antimalarial and an antiprotozoal drug. A search for this DrugBank identifier returns a list of 435 genes from all the 10 nematodes and the two platyhelminthes and is a potential target for repositioning.

**Figure 4:**
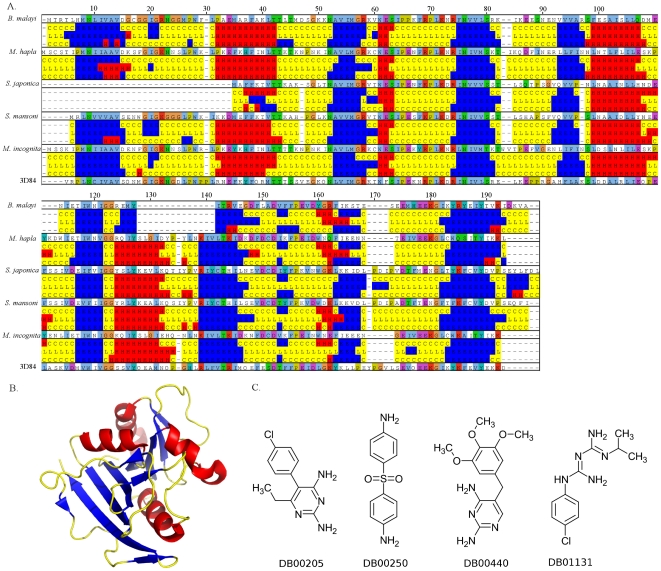
Properties of dihydrofolate reductase, a putative drug target. (A). Alignment of the secondary-structure predictions downloaded from HelmCoP using JalView (Waterhouse et al. 2009) for the following sequences: 15204.m00021 (*B. malayi*), Mh10g200708_Contig1584_9859_10502 (*M. hapla*), Sjc_0071810 (*S. japonica*), Smp_175230 (*S. mansoni*), prot_Minc17846 (*M. incognita*), 3D84 (PDB entry). The sequences are colored based on clustalw (Thompson et al. 1994). For the secondary-structure prediction, helices are colored red, β-sheets blue, and random coils and loops are colored yellow. The fasta from the PDB entry, 3D84, is also shown at the bottom of the alignment. Secondary structure was aligned using a script (provided in the supp information) after aligning fasta sequences provided by HelmCoP using MUSCLE (Edgar 2004). The fasta sequence for the PDB entry was obtained from the PDB website. B. PDB entry 3D84 rendered using Pymol (Version 1.2r3pre) with helices, loops, and β-sheets colored red, yellow, and blue, respectively C. Chemical structures of DrugBank compounds that bind to dihydrofolate reductase targets, which have also been used as antimalarial drugs.

A recent study has found that dihydrofolate reductase (DHFR) inhibitors, which have been used for protozoan parasites, could be used as new antifilarial leads [49]. Using HelmCoP, we searched for the DHFR enzyme, using the KO identifier K00287, for all the parasitic helminthes in HelmCoP: *T. sprialis*, *B. malayi*, *S. mansoni*, *S. japonica*, *M. hapla*, and *M. incognita*, choosing similarity to the PDB, DrugBank and cheminformatic data as output. DHFR targets exist in each genome, and several that have homology to targets for DHFR inhibitors in DrugBank have homology to known PDB structures and have severe RNAi phenotypes. Interestingly, several of these have nematode-specific indels. Using the secondary-structure output from HelmCoP, we demonstrate that many of these proteins share secondary-structure homology to one another (Figure 4A). The fasta sequence for PDB entry 3D84, was aligned to this group using MUSCLE [50] and spans the entire sequence of the nematode dihydrofolate reductase proteins and has similar structural features (Figure 4A and 4B). Some of these proteins also have homology to DrugBank targets and were prioritized using cheminformatic information in HelmCoP; compounds that follow Lipinski's rule of 5 and have less than 8 rotatable bonds. This narrowed the list of compounds from 43 to 24, and out of these 24 compounds, 4 of the drugs have been used as antimalarial drugs (Figure 4C). Homology models could be generated and potential docking studies could be done to suggest modifications to these compounds. SMILES strings for these drugs are also available from HelmCoP and can be used to find similar compounds in publicly available chemical databases based on a Tanimoto similarity score.

Many neurotransmitters and ion-gated channels are good potential drug targets for nematodes [29]. These domains can be searched using their InterPro identifier, and their associated DrugBank targets can be selected as output. By searching HelmCoP for the IPR006201, which is the major group for neurotransmitter-gated ion-channel that includes nicotinic acetylcholine receptor, which has been studied as a potential drug target [51] and gamma-aminobutyric acid A receptor [29], we get a list of 160 genes. Multiple DrugBank targets are returned, including drugs with well-known anthelmintic properties, like ivermectin and piperazine [52].

## Discussion

HelmCoP is a comprehensive database for helminthes for comparative inter- and intra-phylum genomics. Comparative genomic approaches aid in answering fundamental questions regarding parasitism and basic biological aspects that define the pathogen. It is used as a springboard along with extensive supporting information and filters, to prioritize potential drug and vaccine targets for helminthes. The need for drug and pesticide discovery to fight parasitic helminth infections is growing in importance, as resistance to current methods of control has been observed in many nematodes. As more genomes in other phyla get sequenced, HelmCoP can be used as a model resource for combining comparative genomics and drug target discovery.

HelmCoP incorporates functional information including enzymes, compounds and pathways, InterPro domains, Gene Ontology terms, and RNAi phenotype data. Expression data from an extensive collection of ESTs (cDNA and RNAseq data will be included as they become available) derived from multiple stages and tissues, orthology information, secondary-structure prediction, homologies to proteins in the PDB and targets from DrugBank, and protein-protein interactions are also hosted. Search filters implemented for several of these features assist in detecting a specific subset of genes of interest, reducing the number of genes to study for potential drug targets and providing important pieces of information for comparative genomics.

HelmCoP includes an extensive Frequently Asked Questions (FAQ) section that provides detailed information on the search and output parameters and explanations on how they were derived. Nematode.net [10] features a user forum with a section dedicated to HelmCoP. This will allow communication between users and offers a place to facilitate discussions on drug and vaccine prioritization for helminthes.

Several recent studies have examined prioritization of drug targets in parasitic helminthes [53], [54], but these are specific to one species. The TDR database [55] prioritizes drugs and drug targets for many neglected tropical diseases, including protozoan and bacterial parasites; however, TDR only includes one nematode and one platyhelminth species, and has much fewer features compared to HelmCoP. HelmCoP combines 10 nematodes, 2 platyhelminthes, and 6 hosts/outgroups to provide the most comprehensive collection of NTD proteomes available, while also enabling comparison to two plant hosts (*G. max* and *A. thaliana*) and two animal hosts (*M. musculus* and *H. sapiens*).

We plan to include the upcoming helminth genomes [5] into HelmCoP as they become available and to make this resource more functional in the next release (annual releases are planned), by incorporating more search functionality and converting some of the “output only” options to searchable terms. We plan to include searches based on number of transmembrane domains present, number of coiled-coil heptads, PPI interactions, Lipinski's rule of five, and expression profiles. In addition, we also plan to add the TTD database [56] in the next release to enable searches to more therapeutic targets. Enabling the user to filter based on these additional parameters will aid in producing a more defined set of protein targets for experimental testing, as well as provide a more useful tool for comparing nematode genomes.

Sequencing is underway for over 20 helminth genomes, and other data, like transcriptomic sequences, are constantly being generated. This additional data will enable powerful comparative analytic approaches as more species data are added. In future releases, we plan to include other types of data like micro-array based expression and RNA-seq data (e.g. [57]). We also plan to focus on SNP detection and comparison of clinical isolates as they become available. Although the drop in sequencing cost has been substantial in the recent past there are still a lot of helminth species with only partial genomes available. Over 30 parasitic helminthes from different phylogenetic clades have ESTs/cDNA, and these sequences have been assembled into contigs (putative transcripts) and clusters (representing putative genes) [10]. This data will also be included in future releases of HelmCoP to take advantage of all available data and further increase the utility of HelmCoP.

HelmCoP is an important resource for comparative genomics and drug prioritization for helminthes, providing the most comprehensive set of data for the phyla Nematoda and Platyhelminthes currently available. This resource will be updated as new sequencing data becomes available and additional functionalities are currently being considered. The HelmCoP resource has the potential to assist researchers in answering basic biological questions regarding these important pathogens, while also providing a user-friendly interface to prioritize drugs and drug targets, as well as vaccines and pesticides.

## Materials and Methods

### Database design

The design of the database and interface reflects the relational, object-based data models embedded in our analytic pipeline. HelmCoP was designed to be used both for returning large lists of genes matching specified filters along with associated annotations and for returning all related information for a specific gene or function. The results of any type of search are displayed with associated gene features defined by the user, and in addition, a tab-delimited text file is also available for download. Searches can be restricted to specific nematode species, or phylogenetic clades, in addition to reporting results matching a specific annotation, like an interpro domain, KO number, or PDB identifier. The resulting output can be tailored to report only features in which the user is interested.

HelmCoP contains a query engine constructed over a relational database built on MySQL. For keyword searches, a MySQL relational database is used for sequence storage with queries and the display is mediated by Perl CGI scripts. HelmCoP is hosted on the backend by a MySQL server version (v8.41, distribution 5.0.51a), through the Nematode.net [10] portal. HelmCoP has been designed with the GUI-based visual HTML editor Adobe Dreamweaver CS3, combined with an in-house Perl-mediated template-sourcing scheme that is fast, flexible, and allows for code recycling. HelmCoP will be maintained independently of Nematode.net. Two kinds of updates are planned for the HelmCoP databases and resources. First, data not expected to change frequently, such as PairCoil2, secondary structure and disorder predictions, and orthology output, will be updated when a new or improved assembly of the genome, with a more complete proteome becomes available. Other annotations that have new versions released frequently will be updated every six months. We have developed an automated pipeline for performing regularly scheduled updates to the HelmCoP database for these annotations.

### Datasets and Orthology

HelmCoP includes data from 18 organisms that have their proteome available (Table 1). These were obtained from the following locations – All *Caenorhabditis spp.* (wormbase.org, version 204), *Brugia malayi* [58], *Pristionchus pacificus* [59], *Trichinella spiralis* (Mitreva et al. 2011), *Meloidogyne incognita* [60], *Meloidogyne hapla* [61], *Homo sapiens*, *Mus musculus* and *Saccharomyces cerevisiae* (downloaded from http://www.biomart.org; Dec 2009), *Arabidopsis thaliana* (http://www.arabidopsis.org/), *Drosophila melanogaster* (http://flybase.org/), *Glycine max* (http://www.phytozome.net/soybean ), *Schistosoma mansoni* [62], *Schistosoma japonicum* [63]. For genomes where isoforms are known, all isoforms were used. Orthology was determined using ORTHOMCL version 2 using default parameters [13].

### Structure

The following secondary-structure prediction programs were used: JUFO [64], PHD PROF [65], Psipred [66]. Coiled-coil regions were determined using Paircoil2 [67]. Regions of disorder were predicted using RONN [68] and IUPRED [69]. Signal peptides and transmembranes regions were predicted using the Phobius web-server [70]. All programs used default parameters. Indels in a subset of genomes [41], [42] are also associated with the genes (Table S2). Homology to PDB [71] [download on 3-2-10] was determined using WU-BLASTP [12] with an e-value of 1e^−5^.

### Function/Essentiality

IntAct [45] and MINT [44] databases record PPIs in terms of UniProtIDs, so protein names from the proteomes were mapped to UniProtID. PPIs from genomes with the largest numbers of known PPIs were used. Proteins from *A. thaliana*, *S. cerevisae*, *H. sapiens*, *D. melanogaster* were mapped to UniProt identifiers using WU-BLASTP with an e-value cutoff of 1e^−20^. The sequences associated with UniProt were downloaded from http://www.uniprot.org on 9-16-10. To map *C. elegans* and *M. musculus* genes to UniProt names, files indicating associations of protein names to UniProt IDs were downloaded from wormmart204 and biomart (9-17-10), respectively.

Similarity of nematode proteins to KO and EC ids (from KEGGv50) was determined using WU-BLASTP with an e-value cutoff of 1e^−10^. InterProScan [17] was run on all the helminth species to obtain InterProIDs and GO annotation. RNAi phenotype information was obtained for *C. elegans* from wormbase (wormpep205). EST homology for each species was determined by searching against genes from that species using WU-BLAST with an e-value filter of 0.1 and only retaining the top best hit for each EST sequence.

### Drug and Drug Target

Nematode proteins similar to targets in DrugBank [9] were determined using WU-BLASTP with an e-value cutoff of 1e^−5^. Cheminformatic information regarding drug targets was calculated using ChemViz within Cytoscape (using extracted SMILES strings from DrugBank) [72]. To determine if the protein is classified as a “druggable” target, InterPro IDs considered druggable based on [33] criteria were used.

### Vaccine Candidates

Several different motifs are considered epitopes that have potential to be developed into a vaccine [34]. Regions of disorder between 25 and 100 were determined using RONN. The output of Paircoil2 was mined for coiled coils between 3 and 10 heptad repeats. Based on helminth and platyhelminth InterPro domains, zinc-fingers, knottins, animal toxins, EGF modules, and collagens were mined based on a manually curated set of InterPro IDs (Table S5).

## Supporting Information

Table S1 **List of genes and groups from all combinations of ortholog groups from the 18 species.** Numbers under the groups column represent the number of orthologous group from the specific combination of species which is represented by species that have at least one protein under the species columns.
(XLSX)

Table S2 **Species with proteins having nematode-specific indels from two studies (Wang et al. 2009 and Taylor et al. unplublished) included in HelmCoP.**
(XLSX)

Table S3 **Number of ESTs used as evidence for expression of helminth transcripts.**
(XLSX)

Table S4 **An example of comparative genomics search.** (A). Genes from the comparative genomics search for peptidases with signal peptides and severe phenotype. Nematode genes from (B). ortho17taxa3159; (C). ortho17taxa3783; (D). ortho17taxa1084.
(XLSX)

Table S5 **InterPro IDs used as filter for vaccine development.**
(XLSX)

Figure S1 **Comparative genomics of orthologous group, ortho17taxa3783.**
(DOCX)

## References

[1] Mardis ER (2008). The impact of next-generation sequencing technology on genetics.. Trends in Genetics.

[2] Hotez PJ (2010). Nuclear weapons and neglected diseases: the “ten-thousand-to-one gap”.. PLoS Negl Trop Dis.

[3] Kjetland EF, Ndhlovu PD, Gomo E, Mduluza T, Midzi N (2006). Association between genital schistosomiasis and HIV in rural Zimbabwean women.. AIDS.

[4] Hotez PJ, Kamath A (2009). Neglected Tropical Diseases in Sub-Saharan Africa: Review of Their Prevalence, Distribution, and Disease Burden.. PLoS Negl Trop Dis.

[5] Brindley PJ, Mitreva M, Ghedin E, Lustigman S (2009). Helminth genomics: The implications for human health.. PLoS Negl Trop Dis.

[6] Urban JF, Steenhard NR, Solano-Aguilar GI, Dawson HD, Iweala OI (2007). Infection with parasitic nematodes confounds vaccination efficacy.. Vet Parasitol.

[7] Besier B (2007). New anthelmintics for livestock: the time is right.. Trends Parasitol.

[8] Gasbarre LC, Smith LL, Lichtenfels JR, Pilitt PA (2009). The identification of cattle nematode parasites resistant to multiple classes of anthelmintics in a commercial cattle population in the US.. Vet Parasitol.

[9] Wishart DS, Knox C, Guo AC, Cheng D, Shrivastava S (2008). DrugBank: a knowledgebase for drugs, drug actions and drug targets.. Nucleic Acids Research.

[10] Martin J, Abubucker S, Wylie T, Yin Y, Wang Z (2009). Nematode.net update 2008: improvements enabling more efficient data mining and comparative nematode genomics.. Nucleic Acids Res.

[11] von Grotthuss M, Plewczynski D, Ginalski K, Rychlewski L, Shakhnovich EI (2006). PDB-UF: database of predicted enzymatic functions for unannotated protein structures from structural genomics.. BMC Bioinformatics.

[12] WU_BLAST. Available: http://blast.wustl.edu/. Accessed 2006 May 4

[13] Li L, Stoeckert CJ, Roos DS (2003). OrthoMCL: Identification of Ortholog Groups for Eukaryotic Genomes.. Genome Res.

[14] Chen F, Mackey AJ, Vermunt JK, Roos DS (2007). Assessing Performance of Orthology Detection Strategies Applied to Eukaryotic Genomes.. PLoS ONE.

[15] Ashburner M, Ball CA, Blake JA, Botstein D, Butler H (2000). Gene ontology: tool for the unification of biology. The Gene Ontology Consortium.. Nat Genet.

[16] Kanehisa M, Araki M, Goto S, Hattori M, Hirakawa M (2008). KEGG for linking genomes to life and the environment.. Nucl Acids Res.

[17] Hunter S, Apweiler R, Attwood TK, Bairoch A, Bateman A (2009). InterPro: the integrative protein signature database.. Nucleic Acids Res.

[18] Fraser AG, Kamath RS, Zipperlen P, Martinez-Campos M, Sohrmann M (2000). Functional genomic analysis of C. elegans chromosome I by systematic RNA interference.. Nature.

[19] Gonczy P, Echeverri C, Oegema K, Coulson A, Jones SJ (2000). Functional genomic analysis of cell division in C. elegans using RNAi of genes on chromosome III.. Nature.

[20] Maeda I, Kohara Y, Yamamoto M, Sugimoto A (2001). Large-scale analysis of gene function in Caenorhabditis elegans by high-throughput RNAi.. Curr Biol.

[21] Kamath RS, Fraser AG, Dong Y, Poulin G, Durbin R (2003). Systematic functional analysis of the Caenorhabditis elegans genome using RNAi.. Nature.

[22] Rual JF, Ceron J, Koreth J, Hao T, Nicot AS (2004). Toward improving Caenorhabditis elegans phenome mapping with an ORFeome-based RNAi library.. Genome Res.

[23] Sonnichsen B, Koski LB, Walsh A, Marschall P, Neumann B (2005). Full-genome RNAi profiling of early embryogenesis in Caenorhabditis elegans.. Nature.

[24] Mitreva M, Blaxter ML, Bird DM, McCarter JP (2005). Comparative genomics of nematodes.. Trends Genet.

[25] Kalinna BH, Brindley PJ (2007). Manipulating the manipulators: advances in parasitic helminth transgenesis and RNAi.. Trends Parasitol.

[26] Krautz-Peterson G, Bhardwaj R, Faghiri Z, Tararam CA, Skelly PJ (2010). RNA interference in schistosomes: machinery and methodology.. Parasitology.

[27] Mitreva M, McCarter JP, Martin J, Dante M, Wylie T (2004). Comparative Genomics of Gene Expression in the Parasitic and Free-living Nematodes Strongyloides stercoralis and Caenorhabditis elegans.. Genome Res.

[28] McCarter JP, Mitreva MD, Martin J, Dante M, Wylie T (2003). Analysis and functional classification of transcripts from the nematode Meloidogyne incognita.. Genome Biol.

[29] Overington JP, Al-Lazikani B, Hopkins AL (2006). How many drug targets are there?. Nat Rev Drug Discov.

[30] Lipinski CA, Lombardo F, Dominy BW, Feeney PJ (2001). Experimental and computational approaches to estimate solubility and permeability in drug discovery and development settings.. Adv Drug Deliv Rev.

[31] Tice CM (2001). Selecting the right compounds for screening: does Lipinski's Rule of 5 for pharmaceuticals apply to agrochemicals?. Pest Manag Sci.

[32] Weininger D (1988). SMILES, a chemical language and information system. 1. Introduction to methodology and encoding rules.. Journal of Chemical Information & Computer Sciences.

[33] Hopkins AL, Groom CR (2002). The druggable genome.. Nat Rev Drug Discov.

[34] Corradin G, Villard V, Kajava AV (2007). Protein structure based strategies for antigen discovery and vaccine development against malaria and other pathogens.. Endocr Metab Immune Disord Drug Targets.

[35] Villard V, Agak GW, Frank G, Jafarshad A, Servis C (2007). Rapid identification of malaria vaccine candidates based on alpha-helical coiled coil protein motif.. PLoS ONE.

[36] Olugbile S, Kulangara C, Bang G, Bertholet S, Suzarte E (2009). Vaccine potentials of an intrinsically unstructured fragment derived from the blood stage-associated Plasmodium falciparum protein PFF0165c.. Infect Immun.

[37] Han ET, Lee WJ, Sattabongkot J, Jang JW, Nam MH (2010). Sequence polymorphisms of Plasmodium vivax ookinete surface proteins (Pvs25 and Pvs28) from clinical isolates in Korea.. Trop Med Int Health.

[38] Fink AL (2005). Natively unfolded proteins.. Curr Opin Struct Biol.

[39] Shimizu K, Toh H (2009). Interaction between intrinsically disordered proteins frequently occurs in a human protein-protein interaction network.. J Mol Biol.

[40] Cheng Y, LeGall T, Oldfield CJ, Mueller JP, Van YY (2006). Rational drug design via intrinsically disordered protein.. Trends Biotechnol.

[41] Wang Z, Martin J, Abubucker S, Yin Y, Gasser RB (2009). Systematic analysis of insertions and deletions specific to nematode proteins and their proposed functional and evolutionary relevance.. BMC Evol Biol.

[42] Nandan D, Lopez M, Ban F, Huang M, Li Y (2007). Indel-based targeting of essential proteins in human pathogens that have close host orthologue(s): Discovery of selective inhibitors for Leishmania donovani elongation factor-1.. Proteins.

[43] Fletcher S, Hamilton AD (2006). Targeting protein-protein interactions by rational design: mimicry of protein surfaces.. J R Soc Interface.

[44] Chatr-Aryamontri A, Zanzoni A, Ceol A, Cesareni G (2008). Searching the protein interaction space through the MINT database.. Methods Mol Biol.

[45] Aranda B, Achuthan P, Alam-Faruque Y, Armean I, Bridge A (2010). The IntAct molecular interaction database in 2010.. Nucleic Acids Res.

[46] Brooks DR, Appleford PJ, Murray L, Isaac RE (2003). An essential role in molting and morphogenesis of Caenorhabditis elegans for ACN-1, a novel member of the angiotensin-converting enzyme family that lacks a metallopeptidase active site.. J Biol Chem.

[47] Craig H, Isaac RE, Brooks DR (2007). Unravelling the moulting degradome: new opportunities for chemotherapy?. Trends Parasitol.

[48] Frand AR, Russel S, Ruvkun G (2005). Functional genomic analysis of C. elegans molting.. PLoS Biol.

[49] Bag S, Tawari NR, Sharma R, Goswami K, Reddy MV (2010). In vitro biological evaluation of biguanides and dihydrotriazines against Brugia malayi and folate reversal studies.. Acta Trop.

[50] Edgar RC (2004). MUSCLE: multiple sequence alignment with high accuracy and high throughput.. Nucleic Acids Res.

[51] Williamson SM, Robertson AP, Brown L, Williams T, Woods DJ (2009). The nicotinic acetylcholine receptors of the parasitic nematode Ascaris suum: formation of two distinct drug targets by varying the relative expression levels of two subunits.. PLoS Pathog.

[52] Holden-Dye L, Walker RJ (2007). Anthelmintic drugs.. Worm Book.

[53] Doyle MA, Gasser RB, Woodcroft BJ, Hall RS, Ralph SA (2010). Drug target prediction and prioritization: using orthology to predict essentiality in parasite genomes.. BMC Genomics.

[54] Kumar S, Chaudhary K, Foster JM, Novelli JF, Zhang Y (2007). Mining predicted essential genes of Brugia malayi for nematode drug targets.. PLoS ONE.

[55] Aguero F, Al-Lazikani B, Aslett M, Berriman M, Buckner FS (2008). Genomic-scale prioritization of drug targets: the TDR Targets database.. Nat Rev Drug Discov advanced online publication.

[56] Chen X, Ji ZL, Chen YZ (2002). TTD: Therapeutic Target Database.. Nucleic Acids Res.

[57] Hillier LW, Reinke V, Green P, Hirst M, Marra MA (2009). Massively parallel sequencing of the polyadenylated transcriptome of C. elegans.. Genome Res.

[58] Ghedin E, Wang S, Spiro D, Caler E, Zhao Q (2007). Draft genome of the filarial nematode parasite Brugia malayi.. Science.

[59] Dieterich C, Clifton SW, Schuster LN, Chinwalla A, Delehaunty K (2008). The Pristionchus pacificus genome provides a unique perspective on nematode lifestyle and parasitism.. Nat Genet.

[60] Abad P, Gouzy J, Aury J-M, Castagnone-Sereno P, Danchin EGJ (2008). Genome sequence of the metazoan plant-parasitic nematode Meloidogyne incognita.. Nat Biotech.

[61] Opperman CH, Bird DM, Williamson VM, Rokhsar DS, Burke M (2008). Sequence and genetic map of Meloidogyne hapla: A compact nematode genome for plant parasitism.. Proceedings of the National Academy of Sciences.

[62] Berriman M, Haas BJ, LoVerde PT, Wilson RA, Dillon GP (2009). The genome of the blood fluke Schistosoma mansoni.. Nature.

[63] The Schistosoma japonicum Genome Sequencing and Functional Analysis Consortium (2009). The Schistosoma japonicum genome reveals features of host-parasite interplay.. Nature.

[64] Meiler J, Baker D (2003). Coupled prediction of protein secondary and tertiary structure.. Proc Natl Acad Sci U S A.

[65] Rost B, Yachdav G, Liu J (2004). The PredictProtein server.. Nucleic Acids Res.

[66] McGuffin LJ, Bryson K, Jones DT (2000). The PSIPRED protein structure prediction server.. Bioinformatics.

[67] McDonnell AV, Jiang T, Keating AE, Berger B (2006). Paircoil2: improved prediction of coiled coils from sequence.. Bioinformatics.

[68] Yang ZR, Thomson R, McNeil P, Esnouf RM (2005). RONN: the bio-basis function neural network technique applied to the detection of natively disordered regions in proteins.. Bioinformatics.

[69] Dosztanyi Z, Csizmok V, Tompa P, Simon I (2005). IUPred: web server for the prediction of intrinsically unstructured regions of proteins based on estimated energy content.. Bioinformatics.

[70] Kall L, Krogh A, Sonnhammer EL (2007). Advantages of combined transmembrane topology and signal peptide prediction–the Phobius web server.. Nucleic Acids Res.

[71] Berman HM, Westbrook J, Feng Z, Gilliland G, Bhat TN (2000). The Protein Data Bank.. Nucleic Acids Res.

[72] Shannon P, Markiel A, Ozier O, Baliga NS, Wang JT (2003). Cytoscape: a software environment for integrated models of biomolecular interaction networks.. Genome Res.

