# Supplementary material for: HelmCoP: An Online Resource for Helminth Functional Genomics and Drug and Vaccine Targets Prioritization
Source: PLoS One. 2011 Jul 8;6(7):e21832. doi: 10.1371/journal.pone.0021832 (PMC3132748; doi:10.1371/journal.pone.0021832)

Figure S1. Comparative genomics of orthologous group, ortho17taxa3783. A. Truncated output of the NemaCaP search (the complete search can be found in Supplemental Table 4C). Alignment of B. IUPRED and C. RONN disorder prediction programs. The disorder of the following proteins from ortho17taxa3783 were plotted: 14990.m07830 (*B. malayi*), CBG04194 (*C. briggsae*), CBN08355 (*C. brenneri 1*), CBN28391 (*C. brenneri 2*), GENEPREDICTION_SNAP300000061681 (*P. pacificus*), Mh10g200708_Contig31_40190_41290 (*M. hapla*), Y54E10BR.5 (*C. elegans*), prot_Minc04013 (*M. incognita 1*), prot_Minc06190 (*M. incognita 2*). D. Alignment of the secondary-structure predictions downloaded from NemaCaP using JalView [Waterhouse et al. 2009]. The same proteins shown in C. are shown here. The sequences are colored based on clustalw. For the secondary-structure prediction, helices are colored red, β-sheets blue, and random coils and loops are colored yellow. The disorder and secondary structure were aligned using a script (provided in the supp information) after aligning fasta sequences provided by NemaCaP using muscle [Edgar 2004].


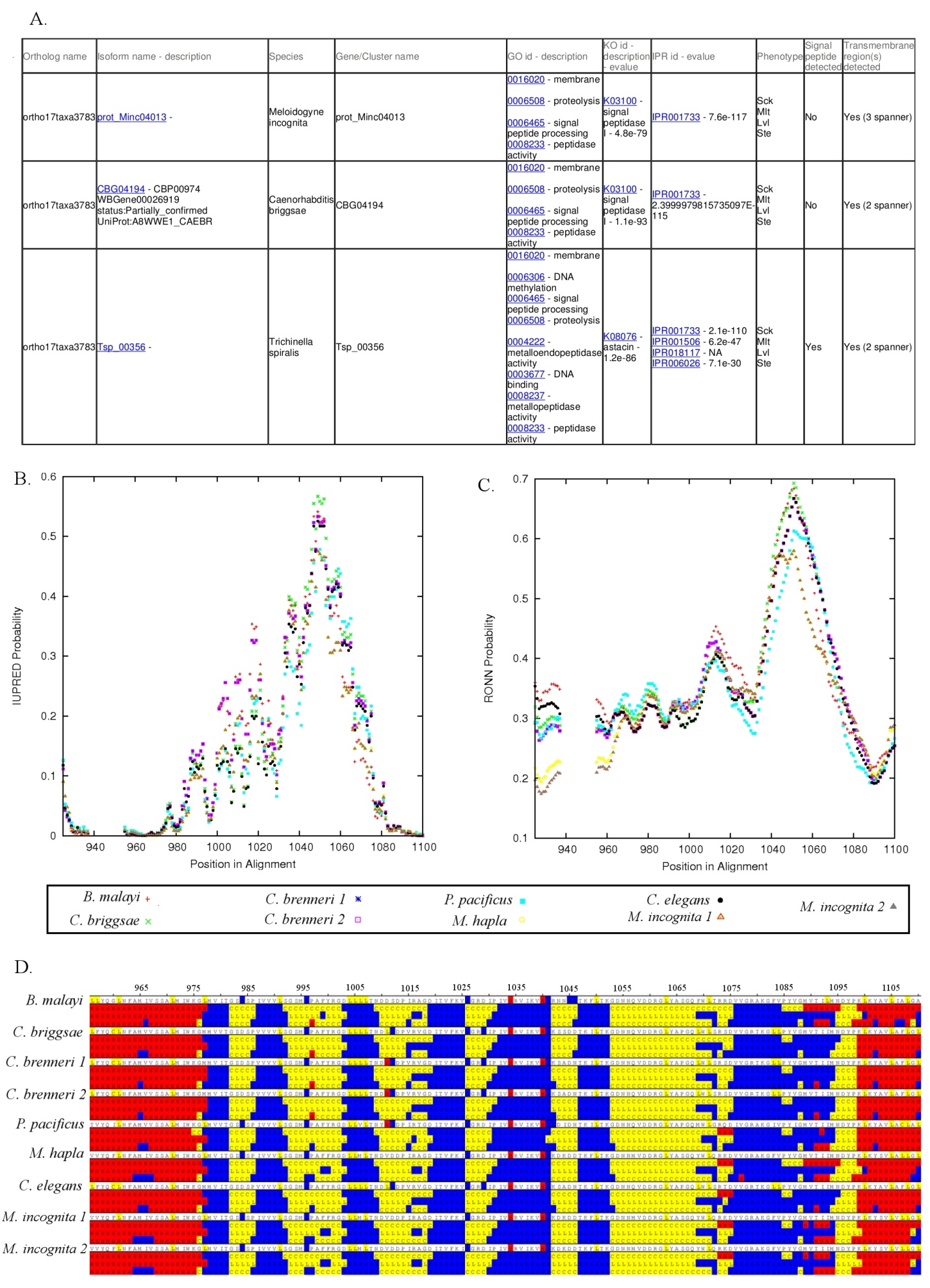

Supplement: Figure S1 — Comparative genomics of orthologous group, ortho17taxa3783. (DOCX) [file pone.0021832.s006.docx]
